# Fat-free mass is associated with exercise pressor responses, but not cold pressor responses, in humans: influence of maximal voluntary contraction

**DOI:** 10.3389/fspor.2024.1352192

**Published:** 2024-03-05

**Authors:** Jon Stavres, Ryan S. Aultman, Caleb F. Brandner, Ta’Quoris A. Newsome, Anabelle Vallecillo-Bustos, Austin J. Graybeal

**Affiliations:** ^1^ School of Kinesiology and Nutrition, The University of Southern Mississippi, Hattiesburg, MS, United States; ^2^ Department of Kinesiology, Iowa State University, Ames, IA, United States

**Keywords:** blood pressure, body mass index, body composition, cardiovascular reflex, handgrip

## Abstract

**Objective:**

This study examined the contributions of fat mass (FM) and fat-free mass (FFM) to the magnitude of exercise pressor responses in humans.

**Methods:**

The cumulative blood pressure responses (blood pressure index; BPI) to handgrip exercise (BPI_hg_), post-exercise-circulatory-occlusion (BPI_peco_), and cold-pressor activation (BPI_cpt_) were collected from 67 individuals grouped by BMI (27.8 ± 7.3 kg/m^2^), FFM index (FFMi, 29.1 ± 3.8 kg/m^2^), and FM index (FMi 12.5 ± 4.8 kg/m^2^) quartiles. BPI responses to HG were also normalized to the time-tension index of HG, providing a relative index of exercise pressor response magnitude (BPI_norm_).

**Results:**

BPI_hg_ and BPI_peco_ were significantly elevated in the third FFMi quartile (*p *≤ 0.034), while BPI_norm_ significantly decreased in the second and fourth quartiles (*p *≤ 0.029). In contrast, no differences in BPI_cpt_ were observed across any FFMi, BMI, or FMi quartiles (*p *≥ 0.268). FFM was independently associated with BPI_hg,_ BPI­_peco_, and BPI_norm_ (all *p *≤ 0.049), however, FFM was eliminated as an independent predictor when maximal voluntary contraction (MVC) was included in these regression models (all *p *≥ 0.495). Neither FFM nor MVC was associated with BPI_cpt_ (*p *≥ 0.229).

**Conclusions:**

These findings indicate that exercise pressor responses, but not cold-pressor responses, are significantly associated with FFM in humans, and that this association is driven by FFM related differences in MVC.

## Introduction

Previous research has demonstrated that individuals with obesity experience a variety of autonomic dysfunctions, including elevated resting sympathetic activity (1) and impaired baroreflex control (2). Some studies have also reported exaggerated exercise pressor responses in both children (3) and young adults with obesity (4), which is characteristic of other obesity-related conditions, including metabolic syndrome (5, 6) and type 2 diabetes (7, 8). Given the association between adiposity and resting sympathetic activity (1, 2), these findings might suggest that the excess adiposity associated with obesity would contribute to these exaggerated exercise pressor responses. However, this relies on the assumption that increases in resting sympathetic activity are associated with augmented exercise pressor responses, which is not always the case. Prior studies in hypertensive individuals have reported exaggerated exercise pressor responses despite no significant differences in muscle sympathetic nerve activity (MSNA) readings at baseline (9, 10), whereas other studies have reported elevated MSNA at baseline despite no significant differences in the overall pressor response (11, 12). Likewise, Park et al. (13) reported significantly elevated MSNA responses to cold pressor activation in individuals who are obese compared to controls, despite no significant differences in the overall blood pressure response. This disassociation between resting MSNA and the blood pressure responses to exercise and cold pressor activation challenges the assumption that obesity related increases in resting sympathetic activity would result in exaggerated exercise pressor responses. Therefore, we must also consider the role of fat-free mass (FFM).

FFM also increases as a function of overall body size, and prior evidence has indicated that the total volume of active muscle mass, a major component of total FFM that is linearly associated with FFM in both obese and non-obese individuals (14), is directly associated with the magnitude of electrically evoked exercise pressor reflex responses in decerebrate rats (15). This evidence suggests that FFM, rather than FM alone, may also contribute to the exaggerated exercise pressor responses observed in obesity. One factor likely to mediate a relationship between FFM and exercise pressor responses is maximal strength. FFM is highly correlated to maximal strength (16), and an increase in maximal strength would reduce the relative workload of a given absolute contraction intensity (i.e., kg force output), thus reducing the central motor drive required to achieve the target contraction intensity (17). This decrease in central motor output and intensity would be associated with a decrease in central command (18, 19), leading to an attenuation of the overall hemodynamic response. Conversely, in the case of a given relative contraction intensity (i.e., % of maximal strength), an increase in FFM may also increase the total volume of active muscle mass required to achieve that force output, resulting in an increased engagement of the exercise pressor reflex as demonstrated by Estrada and colleagues (15), or an increase in central command as demonstrated by Franke et al. (20). However, this assumption is speculative, and no studies have directly evaluated the associations between FM, FFM, and exercise pressor responses in humans.

Accordingly, this study tested the hypothesis that the magnitude of exercise pressor responses would be independently associated with overall FM and FFM in humans, and that the association between FFM and exercise pressor response magnitude would be explained by differences in absolute strength (i.e., MVC). Moreover, considering prior evidence that the hemodynamic responses to post-exercise metaboreflex activation [achieved via post-exercise circulatory occlusion (PECO)] are proportional to absolute work and not relative effort (21), we also hypothesized that the blood pressure responses to PECO would be significantly associated with FFM. Like the pressor responses to handgrip exercise (HG), we expected this relationship to also be explained by MVC. Lastly, to further support the hypothesis that the associations between FFM and exercise pressor and metaboreflex responses would be primarily mediated by differences in central command (secondary to differences in maximal strength) or differences in exercise pressor reflex activation (secondary to FFM related differences in total active muscle mass), we also examined the associations between FFM and the blood pressure responses to the cold-pressor test (CPT). We hypothesized that neither FFM or MVC would be significantly associated with CPT responses, indicating that any observed associations between FFM and exercise pressor or metaboreflex responses would be explained by FFM related differences in central command and/or exercise pressor reflex activation. Likewise, we also expected to observe no associations between FM and CPT responses, consistent with the findings of Park et al. (13).

## Methods

### Experimental design and study participants

This study followed a between-subjects design and required two visits to The University of Southern Mississippi research laboratories. As will be discussed in the following sections, visit 1 consisted of an anthropometric prescreening, while visit 2 consisted of cardiovascular reflex assessments. Seventy-five individuals completed visit 1 of this study protocol, 69 of whom completed visit 2. Two participants were excluded as outliers (mean pressor responses >3.5 standard deviations above the inclusive mean), resulting in a final sample of 67 participants. The final sample was 49.3% male (*n *= 33), 50.7% female (*n *= 34), 92.5% non-Hispanic (*n *= 62), 7.5% Hispanic (*n *= 5), 55.2% White (*n *= 37), 41.8% Black/African American (B/AA; *n *= 28), and 3.0% Asian (*n *= 2). The mean age of the overall sample was 27 ± 10 years, with an average height of 170.7 ± 10.5 cm, an average weight of 82.2 ± 27.1 kg, and an average BMI of 27.8 ± 7.3 kg/m^2^. These 67 participants were subsequently grouped into quartiles for BMI, FFM index (FFMi), and FM index (FMi). Demographics by quartiles are presented in Table 1 and will be discussed further in the results section. Study protocols were approved by The University of Southern Mississippi Institutional Review Board (IRB# 22-1012) and all participants provided written informed consent.

**Table 1:** Participant demographics by quartiles.

		Age (years)	BMI (kg/m^2^)	WC (cm)	BF% (%)	FM (kg)	FFM (kg)	MVC (kg)	SBP (mmHg)	DBP (mmHg)	RHR (bpm)
BMI quartiles											
Mean	Q1 (16)	24	20.7	73.2	24.7	13.7	42.4	34.2	116	71	64
	Q2 (17)	27	24.2	83.5*	26.4	18.2	51.3	40.6	121*	74	62
	Q3 (17)	31	28.4^*†^	96.8^*†^	30.6*	26.0^*†^	60.3*	41.4	119*	72	64
	Q4 (17)	26	37.5^*†‡^	114^*†‡^	35.9^*†^	41.6^*†‡^	73.3^*†‡^	49.5*	126*	74	69
SD	Q1	9	0.935	4.18	5.44	2.63	6.96	11.0	18	10	9
	Q2	11	1.28	5.59	6.76	4.64	10.1	11.7	16	10	6
	Q3	13	1.87	7.39	7.54	5.82	13.1	11.9	17	7	11
	Q4	8	6.96	17.3	6.58	14.7	15.5	18.0	19	11	13
FMi quartiles											
Mean	Q1 (16)	25	22.0	75.9	20.6	12.6	50.3	41.6	120	73	62
	Q2 (17)	27	24.8	85.6	26.4*	19.6	56.0	40.9	121	71	66
	Q3 (17)	30	27.3*	92.5*	31.0^*†^	24.6*	55.7	41.3	118	71	60
	Q4 (17)	26	36.7^*†‡^	114^*†‡^	39.4^*†‡^	42.6^*†‡^	65.8*	42.3	123	76	71^*‡^
SD	Q1	9	1.94	6.36	3.73	2.46	10.4	11.2	22	11	8
	Q2	10	3.91	11.0	3.15	3.69	17.3	15.9	12	7	9
	Q3	13	3.62	10.3	3.10	4.97	14.6	14.5	15	8	8
	Q4	10	7.70	17.5	4.69	13.9	19.0	16.0	21	10	12
FFMi quartiles											
Mean	Q1 (16)	24	23.2	81.3	33.2	20.4	39.6	28.3	112	71	69
	Q2 (17)	30	26.1	87.9	30.2	22.7	48.5*	34.7	114	70	63
	Q3 (17)	28	27.7*	92.2	26.6	23.5	61.3^*†^	46.3^*†^	132	78	61
	Q4 (17)	25	33.9^*†‡^	107^*†^	28.1	33.2*	77.9^*†‡^	56.1^*†‡^	125	72	67
SD	Q1	9	3.65	11.2	6.47	7.25	2.88	4.33	14	8	7
	Q2	13	5.27	14.5	7.58	10.7	6.61	9.82	18	9	6
	Q3	11	5.84	15.2	8.00	11.1	5.93	8.18	13	9	14
	Q4	7	8.93	21.6	8.09	19.0	12.4	13.4	17	9	10

QN quartile group (*sample size*); BMI, body mass index; WC, waist circumference; MVC, maximal voluntary contraction; BF%, body fat percentage; FM, fat mass in kg; FMi, FM index; FFM, fat-free mass in kg; FFM_index_, FFM index; SBP, resting systolic blood pressure; DBP, resting diastolic blood pressure.

* Significantly different from Q1.

^†^ Significantly different from Q2.

^‡^ Significantly different from Q3 (*p *< 0.05). Data presented as mean ± S.D.

### Anthropometrics and body composition assessments

Participants arrived at visit 1 following an overnight fast, including caffeine and over-the-counter medications, and having abstained from intense physical activity (i.e., structured exercise) for at least 24 h prior. Participants were instructed to wear tight fitting athletic clothing, and to secure all hair above the shoulders. Height and weight were measured using a calibrated stadiometer and scale, respectively, and waist circumference (WC) was measured around the iliac crest using a spring-loaded aluminum tape measure according to previously published guidelines (22). Body composition was evaluated using bioimpedance spectroscopy (BIS; SFB7, ImpediMed, Carlsbad, CA), as described elsewhere (6). In brief, BIS provides estimates of total body fat percentage (BF%), FM, and FFM based on estimates of total body water, and has demonstrated validity and reliability in prior human studies (23), including individuals with overweight or obesity (24). This assessment required participants to lay supine for ∼5 min while electrodes placed on the hands and feet delivered an array of electrical currents, and recorded the resistance to those currents. FMi and FFMi were then calculated by dividing FM and FFM, respectively, by height in meters squared.

### Hemodynamic assessments

Participants arrived at visit 2 at least eight hours postprandial, including caffeine, and having abstained from over-the-counter medications for twelve hours and intense physical activity for 24 h prior. All cardiovascular reflex testing was performed in the supine position and began with a two-minute baseline period. Next, participants performed two-minutes of isometric HG of the non-dominant arm, followed by a three-minute period of post-exercise circulatory occlusion (PECO). HG was held constant at 35% of the predetermined maximal voluntary contraction (MVC), which was assessed in triplicate at baseline. PECO was applied to the proximal portion of the arm immediately prior to the end of contraction via a pneumatic pressure cuff (E20 rapid cuff inflator, DE Hokanson, Bellevue, WA). For clarity, HG was employed as a method of eliciting robust exercise pressor responses, which includes the influences of central command (18, 25, 26) and the afferent exercise pressor reflex (27, 28). In contrast, PECO is used as a method of trapping exercise related byproducts within the previously active forearm, thus isolating the metaboreflex component of the exercise pressor reflex. All participants were familiarized with the HG and PECO protocol prior to testing.

As noted previously, the CPT was also used as a non-exercise control stimulus. Each CPT began with a two-minute baseline period, followed by two minutes of ice-water hand immersion and two-minutes of recovery. The ice-water bucket was supported by a table just below the edge of the data collection table at the natural level of the hand. The participant’s hand was then placed in the ice water bucket up to the level of the wrist and maintained for the entire two-minute period. Similar to the HG and PECO trials, participants were familiarized with the CPT protocol prior to testing. All hemodynamic testing was performed in the supine position, and investigators actively encouraged participants to avoid breath-holding and/or Valsalva maneuvers throughout all protocols.

### Data analysis

**Figure 1:**
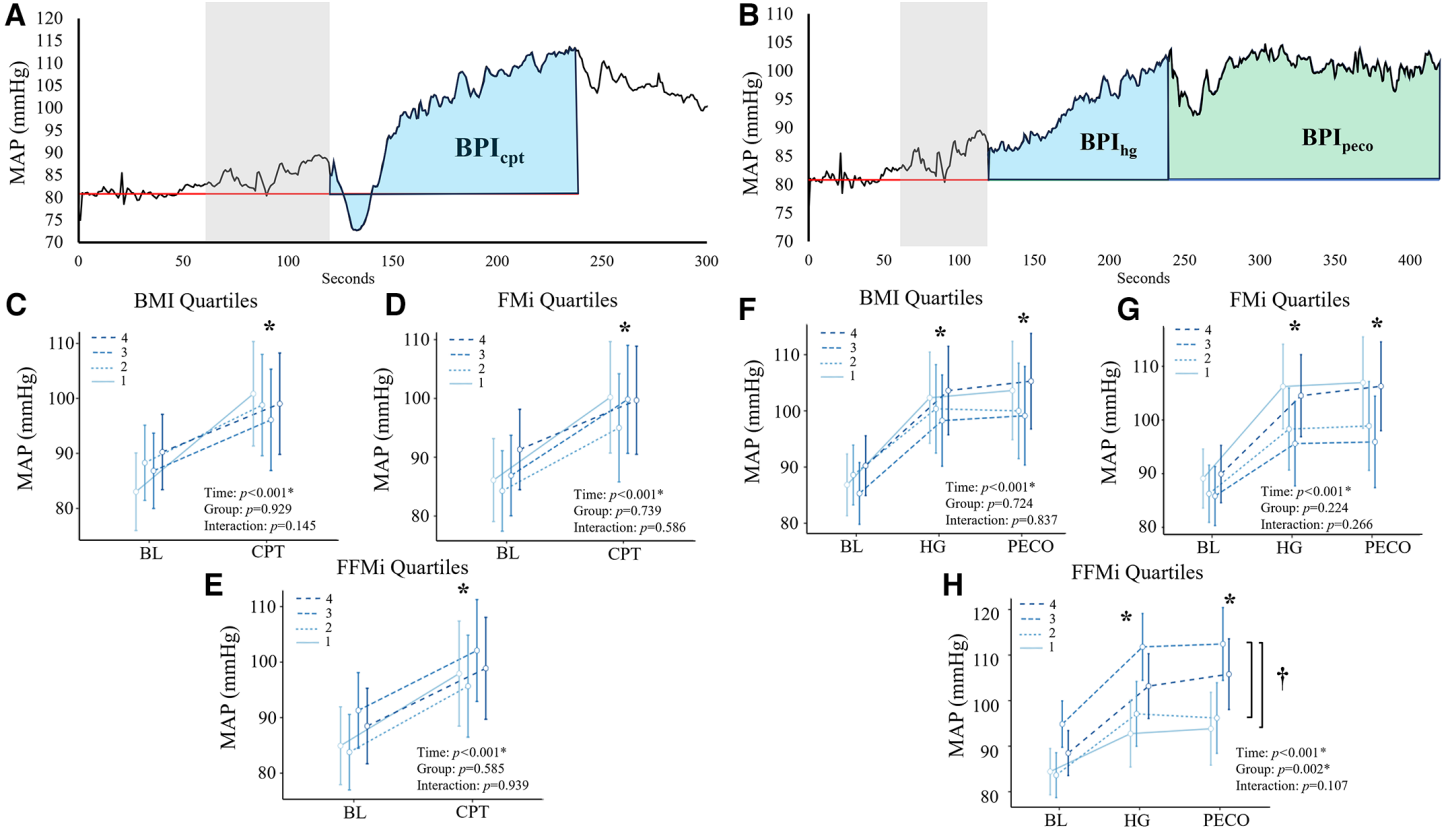
Panels (**A**,**B**) depict the beat-by-beat blood pressure responses to handgrip (HG) and post-exercise circulatory-arrest [PECO; (**A**)] and cold pressor activation (**B**) the blue and green shaded areas represent the calculation of the blood pressure index (BPI) for HG (BPI_hg_), PECO (BPI_peco_), and cold-pressor activation (BPI_cpt_). The gray shaded area represents the final minute of baseline, which was excluded due to anticipatory responses. Panels c-e illustrate the changes in mean arterial pressure (MAP) during cold-pressor activation compared between quartiles for body mass index [BMI; (**C**)], fat mass index [FMi, (**D**)], and fat-free mass index [FFMi, (**E**)]. Likewise, panels f-h illustrate the changes in MAP across HG and PECO compared between BMI quartiles (**F**), FMi quartiles (**G**), and FFMi quartiles (**H**) *, significant main effect of time independent of group; †, significant main effect of group independent of time. Significance accepted at *p *< 0.050.

Throughout the baseline (preceding both HG and CPT), HG, PECO, and CPT time periods, heart rate (HR) was recorded via a one-lead electrocardiogram (lead I) and beat-by-beat blood pressure was recorded via finger photoplethysmography using the non-exercising arm (Finapres NANO; AD Instruments, Colorado Springs, CO). Brachial blood pressures were also recorded prior to each baseline period (Tango M2, SunTech Medical, Morrisville, NC), which were used to calibrate the raw beat-by-beat blood pressure collected during the same time period. HG force (kg) was also recorded in real-time using a standard HG dynamometer (Smedley Hand Dynamometer, Stoelting, Wood Dale, IL) fitted with a potentiometer. HG force, beat-by-beat blood pressure, and HR data were simultaneously recorded at 600–1,000 Hz using a multi-channel data acquisition system (PowerLab, AD Instruments, Colorado Springs, CO), and subsequently stored for offline analysis. Mean arterial pressure (MAP) was then calculated as the average value across all samples within each cardiac cycle. To provide an index of the overall blood pressure responses to HG, PECO, and CPT, a blood pressure index (BPI; mmHg*sec) was calculated as the area under the curve for MAP during each condition (illustrated in Figures 1A,B). Of note, baseline data were averaged over the first minute of the two-minute period, whereas the second minute of baseline was excluded due to anticipatory responses (see Figure 1). Moreover, to provide an index of the magnitude of the exercise pressor response relative to absolute force, BPI during HG (BPI_hg_) was then normalized to the area under the curve for HG force (kg) across the entire HG period, resulting in a normalized BPI_hg_ value (BPI_norm_; mmHg/kg), as reported previously (6, 29).

Of note, data from a subset of these participants (*n *= 26, compared to *n *= 67 in the present study) were included in a previous publication (6) examining differences in the hemodynamic responses to HG and PECO between individuals with and without metabolic syndrome. However, the analyses performed in this study have not been previously reported.

### Statistical analyses

Participants were grouped into quartiles based on BMI, FFMi, and FMi. Prior to grouping, data were evaluated for normality and the presence of outliers was examined via visual inspection of box plots and histograms. As noted previously, two individuals were excluded due to pressor responses that were >3.5 standard deviations above the inclusive group mean. The resulting quartile specific sample sizes can be found in Table 1. To determine if absolute pressor responses were significantly different across BMI, FMi, and FFMi quartiles, the MAP was averaged across the first minute of baseline and the full CPT trial, and then compared across time (baseline vs. CPT) and between groups (BMI, FMi, and FFMi quartiles) using repeated measures analyses of variance (RMANOVA). RMANOVA were also used to compare MAP responses across baseline, HG, and PECO and between quartile groups. Any significant main effects of time, condition, or condition by time interactions were further examined using post-hoc analyses employing the Sidak correction for multiple comparisons.

Next, one-way analyses of covariance (ANCOVA) were used to test for differences in pressor responses across each quartile group (BMI, FFMi, and FMi) while adjusting each model for biological sex and resting mean arterial pressure (rMAP). Any significant main effects were examined further using Sidak post-hoc comparisons.

To describe the relationships between anthropometric variables and pressor responses, a Pearson's correlation matrix was prepared using the following variables: age, BMI, BF%, FM, FFM, rMAP, resting heart rate (RHR), MVC, BPI_hg_, BPI_norm_, BPI_peco_, and BPI_cpt_. Next, linear regression analyses were used to examine the independent associations between FFM and each pressor response variable (identified as “Model 1” in the results). FM was excluded from these analyses because it showed no significant correlation with any pressor response variable in the previously mentioned Pearson's correlation analysis. Next, a second set of regression models (“Model 2”) were used to reexamine the relationships between FFM and each pressor response variable, while also including BMI, rMAP, biological sex, and MVC. All statistical analyses were performed using a combination of SPSS statistical software (SPSS version 28, IBM Statistics, Armonk, NY) and Jamovi version 2.3. Data are presented as either mean ± S.D., mean ± 95% CI, or mean difference [95% CI] (significance accepted at *p *< 0.05).

## Results

### Quartiles analyses

Differences in anthropometrics, blood pressure, and MVC across quartiles are presented in Table 1. BMI, WC, BF%, and FM all significantly increased across the third and fourth quartiles for BMI and FMi (all *p *≤ 0.049), whereas FFM only significantly increased across the third and fourth quartiles for BMI (all *p *≤ 0.014), and was only significantly elevated in the fourth FMi quartile (*p *= 0.037). Likewise, BMI and FFM significantly increased across the third and fourth quartiles for FFMi (all *p *≤ 0.003), while FM was only elevated in the fourth FFMi quartile compared to the first (*p *= 0.033). WC was also significantly elevated in the fourth FFMi quartile compared to the second and first quartile (both *p *≤ 0.006). Notably, MVC significantly increased across FFMi quartiles (4 > 3 > 2; all *p *≤ 0.023), and was significantly higher in the fourth BMI quartile compared to the first (*p *< 0.001), but was not different across any FMi quartiles (all *p *> 0.99). Systolic blood pressure (SBP) was also lower in the first BMI quartile compared to all others (all *p *≤ 0.028), and RHR was higher in the fourth FMi quartile compared to the third and first (both *p *≤ 0.023).

Comparisons of the mean MAP responses across time and quartile group for both CPT and HG and PECO trials are presented in Figure 1. As expected, main effects of time were observed for MAP across all anthropometric quartile comparisons (all *p *< 0.001). Specifically, MAP was significantly elevated compared to baseline during HG across all BMI quartiles {mean diff: 13 mmHg [95% confidence intervals (CI): 10/16], *p *< 0.001}, all FMi quartiles [mean diff: 14 mmHg (95% CI: 10/18), *p *< 0.001], and across all FFMi quartiles [mean diff: 13 mmHg (95% CI: 10/16), *p *< 0.001]. MAP was also significantly elevated during PECO compared to baseline across all BMI quartiles [mean diff: 14 mmHg (95% CI: 10/18), *p *< 0.001], all FMi quartiles [mean diff: 14 mmHg (95% CI: 10/18), *p *< 0.001], and across all FFMi quartiles [mean diff: 14 mmHg (95% CI: 11/18), *p *< 0.001]. Likewise, MAP was significantly elevated during CPT across all BMI quartiles [mean diff: 12 mmHg (95% CI: 8/15), *p *< 0.001], all FMi quartiles [mean diff: 12 mmHg (95% CI: 8/15), *p *< 0.001], and across all FFMi quartiles [mean diff: 12 mmHg (95% CI: 8/15), *p *< 0.001]. A main effect of group was only observed for MAP values compared between FFMi quartile groups (*F*_3,62_ = 5.34, *p *= 0.002; Figure 1H). *Post-hoc* analyses revealed significant differences between the third and first [mean diff: 16 mmHg (95% CI: 4/28), *p *= 0.004] and third and second FFMi quartiles [mean diff: 14 mmHg (95% CI: 2/26), *p *= 0.013], independent of time point. No significant group by time interactions were observed for any quartile comparison (all *p *≥ 0.107).

**Figure 2:**
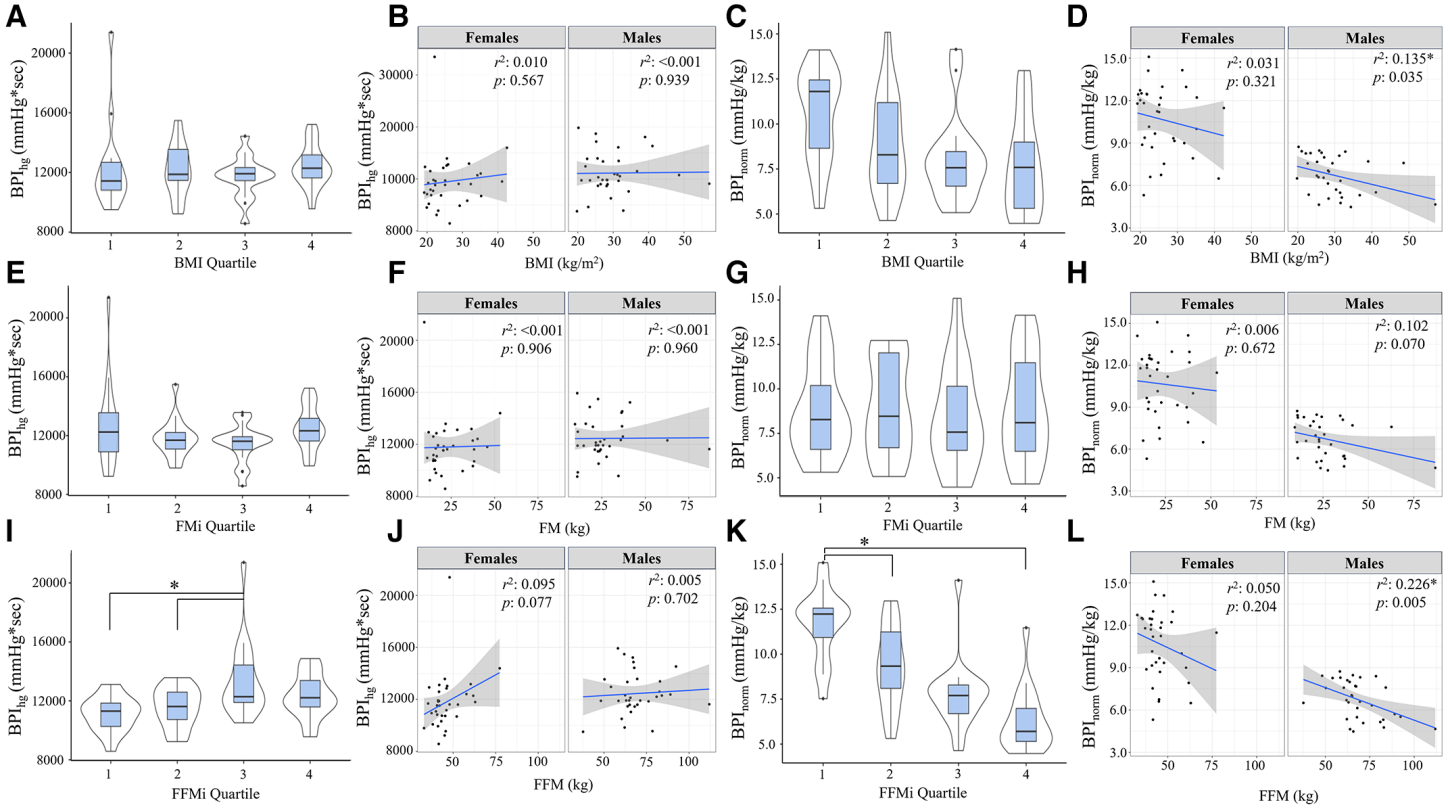
Blood pressure index (BPI) responses during handgrip exercise (BPI_hg_) compared across body mass index quartiles [BMI; (**A**)], fat mass index quartiles [FMi; (**E**)], and fat-free mass index quartiles [FFMi; (**I**)]. Panels (**B**,**F**,**J**) depict the sex-specific associations between BMI and BPI_hg_, FM and BPI_hg_, and FFM and BPI_hg_, respectively. Panels (**C**,**G**,**K**) depict the differences in BPI_hg_ normalized to time-tension index (BPI_norm_) compared between BMI quartiles (**C**), FMi quartiles (**G**), and FFMi quartiles (**K**). Likewise, panels (**D**,**H**,**I**) depict the sex-specific associations between BMI and BPI_norm_, FM and BPI_norm_, and FFM and BPI_norm_, respectively. Brackets indicate significant difference between quartiles, and gray shaded areas represent 95% confidence intervals. Significance accepted at *p *< 0.050.

**Figure 3:**
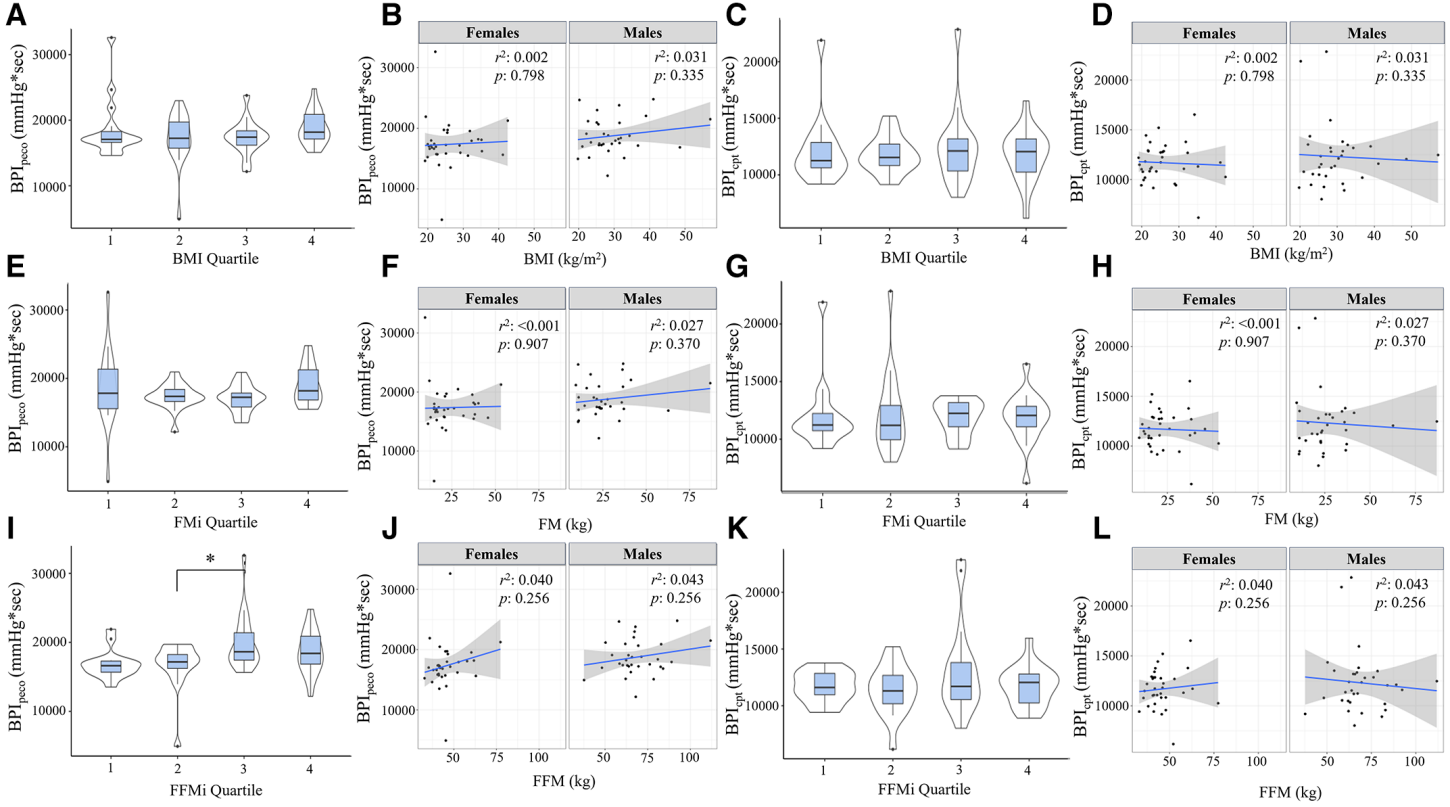
Blood pressure index (BPI) responses during post-exercise circulatory-occlusion (BPI_peco_) compared across body mass index quartiles [BMI; (**A**)], fat mass index quartiles [FMi; (**E**)], and fat-free mass index quartiles [FFMi; (**I**)]. Panels (**B**,**F**,**J**) depict the sex-specific associations between BMI and BPI_peco,_ FM and BPI_peco_, and FFM and BPI_peco_, respectively. Panels (**C**,**G**,**K**) depict the differences in BPI responses to cold-pressor activation (BPI_cpt_) compared between BMI quartiles (**C**), FMi quartiles (**G**), and FFMi quartiles (**K**). Likewise, panels (**D**,**H**,**L**) depict the sex-specific associations between BMI and BPI_cpt,_ FM and BPI_cpt_, and FFM and BPI_cpt_, respectively. Brackets indicate significant difference between quartiles, and gray shaded areas represent 95% confidence intervals. Significance accepted at *p *< 0.050.

Comparisons of BPI responses to HG, PECO (BPI_peco_), and CPT (BPI_cpt_) are presented in Figures 2, 3, as are sex-specific associations for each raw anthropometric value and specific hemodynamic value. As illustrated in Figure 1, neither BPI_hg_ or BPI_norm_ were significantly different across any BMI or FMi quartile (all *p *≥ 0.176), and BMI was only significantly and inversely associated with BPI_norm_ in male participants (*r*^2^ = 0.135, *p *= 0.035). In contrast, BPI_hg_ was significantly elevated in the third FFMi quartile compared to the first and second (both *p *≤ 0.004), and BPI_norm­_ was significantly lower in the second and fourth FFMi quartiles compared to the first (both *p *≤ 0.0029). FFM also demonstrated a significant inverse association with BPI_norm_ in male participants (*r*^2 ^= 0.226, *p *= 0.005). As illustrated in Figure 3, neither BPI_peco_ or BPI_cpt_ were significantly different across any BMI or FMi quartile groups (all *p *≥ 0.202), and no significant sex-specific associations were observed between either BPI value and BMI or FM (all *p *≥ 0.335). BPI_peco_ was significantly elevated in the third FFMi quartile compared to the second (*p *= 0.034), but no differences were observed for BPI_cpt_ across FFMi quartiles (all *p *≥ 0.268), nor were any significant sex-specific associations observed between either BPI value and FFM (all *p *= 0.256). Lastly, to determine if the differences in BPI_hg_, BPI_norm_, and BPI_peco_ across FFMi quartiles could be explained by differences in MVC (as hypothesized), these FFMi ANCOVA were rerun with MVC included as an additional covariate. These models produced no significant differences in BPI_norm_ or BPI_peco_ across any FFMi quartile (all *p *≥ 0.075), but BPI_hg_ responses remained significantly elevated in the third FFMi quartile compared to the first and second (*F *= 5.308, *p *≤ 0.011).

### Linear regression analyses

Results from the Pearson's correlation matrix are presented in Table 2. In brief, BMI was only significantly correlated with BPI_norm_ (*r *= −0.343, *p *= 0.004), while FFM was significantly correlated with BPI_hg_ (*r *= 0.242, *p *= 0.049), BPI_norm_ (*r *= −0.648, *p *< 0.001), BPI_peco_ (*r *= 0.274, *p *= 0.026), but not BPI_cpt_ (*r *= 0.064, *p *= 0.609). rMAP was significantly correlated with BPI_norm_ (*r *= −0.279, *p *= 0.022), BPI_peco_ (*r *= 0.253, *p *= 0.040), and BPI_cpt_ (*r *= 0.295, *p *= 0.015); and MVC was significantly correlated with BPI_hg_ (*r *= 0.372, *p *= 0.002), BPI_norm_ (*r *= −0.801, *p *< 0.001), and BPI_peco_ (*r *= 0.366, *p *< 0.001). FM was not significantly associated with any BPI value (all *p *≥ 0.114), and therefore was not included in the regression models.

**Table 2:** Correlation matrix.

		Age	Sex	BMI	FM	FFM	rMAP	MVC	BPI_hg_	BPI_norm_	BPI_peco_	BPI_cpt_
Age (years)	*r*	—										
	*p*	—										
Sex	*r*	0.081	—									
	*p*	0.513	—									
BMI (kg/m^2^)	*r*	−0.007	0.259*	—								
	*p*	0.958	0.034	—								
FM (kg)	*r*	−0.032	0.113	0.949*	—							
	*p*	0.800	0.361	<.001	—							
FFM (kg)	*r*	0.057	0.700*	0.763*	0.643*	—						
	*p*	0.645	<.001	<.001	<.001	—						
rMAP (mmHg)	*r*	0.199	0.323*	0.328*	0.250*	0.406*	—					
	*p*	0.106	0.008	0.007	0.041	<.001	—					
MVC (kg)	*r*	−0.067	0.709*	0.402*	0.261*	0.742*	0.367*	—				
	*p*	0.592	<.001	<.001	0.033	<.001	0.002	—				
BPI_hg_ (mmHg*sec)	*r*	−0.014	0.173	0.099	0.034	0.242*	0.230	0.372*	—			
	*p*	0.912	0.161	0.424	0.787	0.049	0.061	0.002	—			
BPI_norm_ (mmHg*sec)	*r*	−0.005	−0.710*	−0.343*	−0.195	−0.648*	−0.279*	−0.801*	0.030	—		
	*p*	0.965	<.001	0.004	0.114	<.001	0.022	<.001	0.812	—		
BPI_peco_ (mmHg*sec)	*r*	−0.046	0.201	0.150	0.108	0.274*	0.253*	0.366*	0.836*	−0.005	—	
	*p*	0.715	0.105	0.229	0.388	0.026	0.040	0.003	<.001	0.966	—	
BPI_cpt_ (mmHg*sec)	*r*	0.055	0.121	−0.017	−0.041	0.064	0.295*	0.086	0.246*	−0.023	0.206	—
	*p*	0.661	0.329	0.891	0.742	0.609	0.015	0.489	0.045	0.851	0.096	—

BMI, body mass index; FM, fat mass; FFM, fat-free mass; rMAP, resting mean arterial pressure; MVC, maximal voluntary contraction, BPI_hg_, blood pressure index during handgrip; BPI_peco_, blood pressure index during post-exercise cuff occlusion; BPI_norm_, blood pressure index normalized to handgrip force time-tension index; BPI_cpt_, blood pressure index during cold pressor text.

* *p* < .05.

Results from the linear regression analyses are presented in Table 3. In model 1, which included FFM as the sole predictor variable, FFM was significantly associated with BPI_hg_ (*r*^2^ = 0.058, *p *= 0.049), BPI_norm_ (*r*^2 ^= 0.419, *p *< 0.001), and BPI_peco_ (*r*^2 ^= 0.075, *p *= 0.026), but not BPI_cpt_ (*r*^2 ^= 0.004, *p *= 0.609). A second regression model was prepared including FFM, BMI, rMAP, biological sex, and MVC as predictor variables. This omnibus model was improved with the addition of these covariates for BPI_hg_ (*r*^2 ^= 0.182, *p *= 0.028) and BPI_norm_ (*r*^2 ^= 0.686, *p *< 0.001), but not BPI_peco_ (*r*^2 ^= 0.164, *p *= 0.051) or BPI_cpt_ (*r *= 0.104, *p *= 0.229). However, in model 2, FFM was no longer an independent predictor for any BPI value (all *p *≥ 0.495), and instead, MVC became the most prominent independent predictor of both BPI_hg_ (*β* = 0.449, *p *= 0.023) and BPI_norm_ (*β* = −0.617, *p *< 0.001). Biological sex was also a significant independent predictor of BPI_norm_ (*β* = −0.331, *p *= 0.013). Multicollinearity was also evaluated using a variance inflation factor, all of which were less than 10.

**Table 3:** Linear regression analyses.

		BPI_hg_			BPI_norm_			BPI_peco_			BPI_cpt_		
	VIF	*r* ^2^	*β*	*p*	*r* ^2^	β	*p*	*r* ^2^	β	*p*	*r* ^2^	β	*p*
Model 1	–	0.058	–	0.049*	0.419	–	<0.001*	0.075	–	0.026*	0.004	–	0.609
*FFM*	–	–	0.242	0.049*	–	−0.648	<0.001*	–	0.274	0.026*	–	0.064	0.609
Model 2		0.182	–	0.028*	0.686	–	<0.001*	0.164	–	0.051	0.104	–	0.229
*FFM*	8.29	–	0.229	0.495	–	0.099	0.634	–	0.183	0.592	–	0.031	0.929
*BMI*	3.89	–	−0.226	0.326	–	−0.100	0.481	–	−0.138	0.556	–	−0.147	0.541
*RMAP*	1.23	–	0.141	0.275	–	0.048	0.548	–	0.151	0.252	–	0.326	0.018*
*Sex*	3.23	–	−0.292	0.166	–	−0.331	0.013*	–	−0.203	0.341	–	0.061	0.781
*MVC*	2.77	–	0.449	0.023*	–	−0.617	<0.001*	–	0.375	0.061	–	−0.041	0.840

BMI, body mass index; FFM, fat-free mass; FM, fat mass; BPI, blood pressure index; BPI_norm_, blood pressure index relative to time-tension index; PECO, post-exercise-circulatory-occlusion; HG, handgrip exercise; β; unstandardized beta coefficient; VIF, variance inflation factor.

* *p *< 0.05.

## Discussion

This study tested the hypotheses that FM and FFM would both be independently associated with exercise pressor and metaboreflex responses in humans, and that MVC would mediate these relationships. The findings of this study support the hypotheses related to FFM, but not FM. Specifically, the absolute magnitude of exercise pressor (Figure 2) and metaboreflex responses (Figure 3) increased in the third FFMi quartile, but were unchanged across FMi and BMI quartiles. Likewise, exercise pressor responses normalized to work (BPI_norm_; Figure 2) significantly decreased as a function of FFMi quartile, but were unchanged across any FMi or BMI quartile. When examined further, FFM demonstrated a significant and positive association with BPI_hg_ and BPI_peco_, and a significant inverse association with BPI_norm_, all of which were eliminated after including MVC within the regression model (Table 3). However, FM was not correlated with any pressor response variable. Ultimately, these findings indicate that FFM, and not FM, functions as a primary driver of exercise pressor and metaboreflex responses, and that this relationship is largely explained by FFM related differences in MVC. Moreover, the fact that CPT responses were not different across any FFMi quartile and showed no association with FFM or MVC supports the notion that these relationships are mediated by differences in central command and/or exercise pressor reflex activation. This will be discussed further in the following sections, beginning with a review of cardiovascular dysfunction in obesity-related conditions.

### Cardiovascular dysfunction in obesogenic conditions

Many obesity-related comorbidities are independently associated with cardiovascular dysfunction. Hypertension, which has an estimated prevalence of 65%–78% in individuals with obesity (30), is independently associated with impaired endothelial function (31), exaggerated sympathetic activity (9, 10), and impaired baroreflex control (32). Likewise, sympathetic responses to voluntary exercise and exercise pressor reflex activation are known to be exaggerated in both human (8) and animal (7, 33) models of type 2 diabetes, and evidence suggests that blood pressure responses are exaggerated during handgrip exercise in individuals with hypercholesterolemia (34). Moreover, chronic elevations in blood cholesterol are known to contribute to atherosclerotic diseases, some of which have been directly associated with exaggerated sympathetic responses to exercise (35, 36). Taken together, comorbid hypertension, insulin resistance and hyperglycemia, hypercholesterolemia, and development of atherosclerosis are all likely mechanisms by which exercise pressor responses would be altered in obese states. However, as noted above, this study found no association between FM and exercise pressor or metaboreflex responses, and instead, identified FFM related differences in MVC as the primary factor driving differences in these hemodynamic responses. The following section will discuss the likely factors mediating this relationship.

### Association between FFM and hemodynamic responses to exercise

Despite known associations between adipose tissue and resting sympathetic activity (1, 2), the primary finding of this study was that exercise pressor and metaboreflex responses were significantly associated with FFM, and that this relationship was largely mediated by FFM related differences in MVC. Therefore, muscular strength is an important contributing factor, and must be discussed. Prior evidence indicates that increases in FFM correlate strongly to increases in both muscle cross sectional area (CSA) (37) and strength (16). Therefore, while FFM is technically comprised of a wide variety of non-adipose tissues, changes in FFM are considered valuable proxies for overall muscle mass (14). With this in mind, it may be possible that the increase in muscle mass associated with higher levels of FFM likely resulted in decreased activation of central command, a feed-forward pathway contributing to increases in blood pressure during voluntary muscle contractions (38). This could be explained by a few potential mechanisms. First, MVC increased across FFMi quartiles (Table 1), meaning that the same absolute force output would represent a lower relative intensity in individuals with higher FFMi. Prior studies demonstrate that relative intensity functions as a primary driver of overall exercise pressor responses (39), likely owing to an overall decrease in central command. Therefore, one likely explanation for these findings is a decrease in relative effort in individuals with greater FFM, leading to decreases in central command. However, given the fact that BPI_hg_ responses remained significantly elevated in the third FFMi quartile after covarying for MVC, central command may not be the only contributing factor.

The influence of the exercise pressor reflex is also important to discuss. As noted previously, Estrada and colleagues found that the magnitude of the exercise pressor reflex is directly proportional to the volume of muscle mass stimulated during exercise (15). Considering the strong associations between FFM and muscle mass (14) and FFM and strength (16), we would anticipate individuals with more FFM to produce more overall force (as illustrated in Table 1) and exhibit a larger overall blood pressure response, secondary to an increased metabolic perturbation. Likewise, a recent study by Lee and colleagues (21) also found that inducing muscular fatigue via prior eccentric exercise resulted in significant reductions in metaboreflex responses following isometric knee extensor exercise, despite consistent relative exercise intensities. These findings also support a proportional relationship between absolute force and metaboreflex responses, indicating that a higher absolute force output would result in a greater overall metaboreflex response. These findings could very well explain the (1) elevated BPI_hg_ and BPI_peco_ responses observed across FFMi quartiles (Figures 2, 3), (2) the significant associations between FFM, MVC, and handgrip and PECO responses, and (3) the fact that BPI_hg_ responses remained significantly elevated in the third FFMi quartile even after accounting for MVC. However, if the magnitude of the exercise pressor reflex is truly proportional to the volume of active muscle mass, we would also expect these differences across FFMi quartiles and associations with FFM to be abolished after normalizing to force output (BPI_norm_). Considering that we observed both a significant decrease in BPI_norm_ across FFMi quartiles (Figure 1) and a significant inverse association between FFM and BPI_norm_ (Table 3), a decrease in central command secondary to a reduced relative level of effort cannot be excluded. Considering these findings, it seems likely that both factors, a FFM related augmentation of exercise pressor reflex engagement combined with a FFM related decrease in central command, contribute to these relationships.

### Remaining questions regarding adiposity and cardiovascular dysfunction

Historically, adipocytes have been regarded as deposit sites for triglycerides and other lipid components. However, more recent evidence indicates that adipocytes integrate with the sympathetic nervous system (SNS), with several studies implicating a role of leptin in augmenting sympathetic activity (40–42). Interestingly, sympathetic denervation of white adipose tissue has been shown to increase adiposity, lending support to the idea that the SNS may play a pivotal role in lipolysis (43). However, despite this self-regulating pathway, the increase in SNS activity associated with adiposity (1, 2) also likely contributes to furthering cardiovascular dysfunction. However, it is also important to recognize the previously reported inverse association between resting MSNA and exercise pressor responses (44), challenging the notion that increased SNS activity alone would result in exaggerated pressor responses to exercise. More research is needed to better understand how adiposity related increases in SNS activity influence the hemodynamic responses to muscular contractions.

Regarding the exercise pressor reflex, few studies have directly examined changes in obesity independent of comorbid conditions (such as hypertension or metabolic syndrome). Several studies have examined the role of transient potential receptor potential vanilloid 1 (TRPV1) channels in obesity using knockout models (45–47), and generally indicate that TRPV1 channels differentially contribute to thermogenic and sympathetic regulation in animal models of obesity. However, upon review, no studies have directly examined how potential changes in TRPV1 expression or function contribute to obesity-related changes in exercise pressor reflex activation, nor is there an appreciable body of evidence evaluating obesity related changes in the expression or function of acid-sensing ion channels (ASICs) and/or purinergic receptor contributions to exercise pressor reflex engagement. One study did find that potassium-channel mediated vasodilatory responses to stimulated contractions were attenuated in obese Zucker rats (48), and adipocytes can further influence cardiovascular dysfunction via oxidative stress, which is known to contribute to endothelial dysfunction (49). Ultimately, this gap represents an important area of future investigation, as understanding the influence of adiposity and obesity on exercise pressor reflex function will likely improve our understanding of the pathogenesis of cardiovascular dysfunction in obese states.

### Limitations

There are aspects of this study that warrant further consideration. One of the primary limitations of this study is the absence of MSNA or EMG data. EMG data would permit the evaluation of muscular fatigue, or at the very least, provide an index of changes in central motor drive and central command during fatiguing handgrip exercise. Likewise, MSNA data could have been used to provide an index of total sympathetic drive during handgrip and PECO, further elucidating potential differences in vascular responses. We also did not collect indices of perceived effort during the handgrip protocol (i.e., ratings of perceived exertion), nor did we measure byproducts of muscle metabolism (i.e., lactate concentrations). Both of these measures would have provided further insight into the level of central command and metaboreflex activation, respectively. We also did not collect segmental body composition or forearm limb volume in participants, and therefore, this study relies on the assumption that FFM distribution is consistent between the whole-body and the forearm across subjects. Similarly, while BIS has demonstrated validity and reliability in individuals with and without obesity (23, 24), there still exists inherent variation on a subject-by-subject level that may contribute to increases in overall variance. However, given the fact the influence of FFM on pressor responses was consistently demonstrated across all analyses, we remain confident in the overall interpretation of these findings. Lastly, menstrual cycle phase was not controlled for in this study, introducing additional variance into the statistical analyses.

## Conclusions

The findings of this study indicate exercise pressor responses in humans are primarily driven by FFM, as opposed to FM or BMI. Specifically, with higher FFM and higher MVC, blood pressure responses to relative effort (i.e., BPI_hg_) are augmented, while blood pressure responses normalized to total work (i.e., BPI_norm_) are attenuated. Likewise, pressor responses to handgrip are significantly elevated as a function of FFMi, even when MVC is included as a covariate. These findings suggest that these relationships between FFM and exercise pressor and metaboreflex responses are driven by a combination of FFM related differences in both central command and exercise pressor reflex function. The lack of association between CPT responses and FFM or MVC further support this notion by ruling out generalized sympathetic overexcitability as a mediating factor. Future research should consider FFM and MVC as potential covariates in studies examining blood pressure responses to exercise in humans, particularly cross-sectional observational studies.

## Data Availability

The raw data supporting the conclusions of this article will be made available by the authors, without undue reservation.

## What is already known about this subject?

• Exercise pressor responses are altered in individuals with obesity and obesity-related chronic diseases.
• Both fat mass and fat-free mass may be associated with the magnitude of exercise pressor responses, but this has yet to be directly examined in humans.

## What are the new findings of this manuscript?

• Fat free mass is significantly associated with the magnitude of exercise pressor responses to handgrip and metaboreflex activation, but not cold-pressor activation.
• These associations are explained by fat free mass related differences in maximal voluntary contraction.

## How might your results change the direction of research or the focus of clinical practice?

• These findings highlight the role of fat free mass and muscular strength in driving exercise pressor responses.
• These findings may be particularly useful for future studies examining changes in fat-free mass associated with periods of weight loss.
